# Vascular Drug Delivery Using Carrier Red Blood Cells: Focus on RBC Surface Loading and Pharmacokinetics

**DOI:** 10.3390/pharmaceutics12050440

**Published:** 2020-05-09

**Authors:** Patrick M. Glassman, Carlos H. Villa, Anvay Ukidve, Zongmin Zhao, Paige Smith, Samir Mitragotri, Alan J. Russell, Jacob S. Brenner, Vladimir R. Muzykantov

**Affiliations:** 1 Department of Systems Pharmacology and Translational Therapeutics, Perelman School of Medicine, University of Pennsylvania; Philadelphia, PA 19104, USA; carlos.h.villa@outlook.com (C.H.V.); jacob.brenner@pennmedicine.upenn.edu (J.S.B.); 2 John A. Paulson School of Engineering and Applied Sciences, Harvard University, Cambridge, MA 02138, USA; anvay_ukidve@g.harvard.edu (A.U.); zmzhao@g.harvard.edu (Z.Z.); mitragotri@seas.harvard.edu (S.M.); 3 Wyss Institute for Biologically Inspired Engineering, Harvard University, Cambridge, MA 02138, USA; 4 Disruptive Health Technology Institute, Carnegie Mellon University, Pittsburgh, PA 15213, USA; pnsmith@andrew.cmu.edu (P.S.); alanrussell@cmu.edu (A.J.R.); 5 Department of Biomedical Engineering, Carnegie Mellon University, Pittsburgh, PA 15213, USA; 6 Department of Biological Sciences, Carnegie Mellon University, Pittsburgh, PA 15213, USA; 7 Department of Chemical Engineering, Carnegie Mellon University, Pittsburgh, PA 15213, USA; 8 Department of Medicine, Division of Pulmonary, Allergy, and Critical Care Medicine, Perelman School of Medicine, University of Pennsylvania, Philadelphia, PA 19104, USA

**Keywords:** red blood cells, drug delivery, pharmacokinetics

## Abstract

Red blood cells (RBC) have great potential as drug delivery systems, capable of producing unprecedented changes in pharmacokinetics, pharmacodynamics, and immunogenicity. Despite this great potential and nearly 50 years of research, it is only recently that RBC-mediated drug delivery has begun to move out of the academic lab and into industrial drug development. RBC loading with drugs can be performed in several ways—either via encapsulation within the RBC or surface coupling, and either ex vivo or in vivo—depending on the intended application. In this review, we briefly summarize currently used technologies for RBC loading/coupling with an eye on how pharmacokinetics is impacted. Additionally, we provide a detailed description of key ADME (absorption, distribution, metabolism, elimination) changes that would be expected for RBC-associated drugs and address unique features of RBC pharmacokinetics. As thorough understanding of pharmacokinetics is critical in successful translation to the clinic, we expect that this review will provide a jumping off point for further investigations into this area.

## 1. Introduction

The idea of using red blood cells (RBC) as carriers for drug delivery initially emerged about half a century ago as an approach to improve enzyme replacement therapy [1]. However, the outbreak of blood-transmitted infections in the 1980s effectively halted progress in the area of RBC-mediated drug delivery. For several decades, this line of investigation was overshadowed by other constituencies of the research enterprise encompassing the design of drug delivery systems (DDS)—liposomes, antibody-drug conjugates, and polymeric nanocarriers, to name a few.

Nevertheless, approaches to use RBCs as carriers for pharmacological agents have recently gained significant and rapidly growing attention. Progress in this field is rapidly diversifying and accelerating towards potentially clinically useful products. Many groups are now investigating the use of RBCs in drug delivery and are making significant contributions, leading to breakthrough findings and upbeat investments. Several RBC-based drug delivery approaches have entered clinical trials, including RBC-encapsulated asparaginase (Erytech, Phase 3) and dexamethasone (EryDel, Phase 3). Novel advanced strategies are emerging, including genetic molecular modifications of RBC [2,3], modulation of the immune system by RBC-coupled antigens [3,4], and vascular transfer of RBC-coupled nanocarriers (RBC hitchhiking) [5,6,7].

Both encapsulation into and coupling to the surface of RBC fundamentally transform the key parameters of absorption, distribution, metabolism, and elimination (ADME) of drugs and drug delivery systems (DDS), including diverse nanocarriers. To our knowledge, studies of the pharmacokinetics (PK) and pharmacodynamics (PD) of RBC-based DDS are lacking, despite great relevance for industrial development and clinical utility.

In order to help to close this gap of knowledge, in this paper we undertook the first attempt to define specific, salient parameters controlling behavior of RBC/DDS in the body. Our goal is to provide the modular framework for experimental and theoretical pre-clinical and clinical investigations of ADME-PK-PD features of RBC-based drug delivery.

## 2. Principles of RBC Drug Delivery

### 2.1. Encapsulation of Drugs into Carrier RBC

Loading drugs into the carrier RBC at the present time can be achieved only in isolated RBCs. The most advanced approach involves osmotic swelling, causing transient pores in the RBC membrane (see below). Novel experimental approaches include attempts to use cell-penetrating peptides to import therapeutic proteins in the carrier RBC [8] and fusion of RBC with drug-loaded liposomes [9].

Drug encapsulation into RBCs for use in humans is currently achieved either in vitro or ex vivo using either autologous blood or matching donor blood as a source for RBCs. Washed RBCs are loaded with drugs via transient pores formed in the membrane of RBC during osmotic swelling in hypotonic buffer containing a high concentration of drugs, with subsequent washing with an excess amount of drug [10,11]. Notably, this process does release some hemoglobin from the RBCs [10,11]. The typical procedure, for example, using a semi-automatic device developed by EryDel takes about an hour [12], after which washed and loaded RBCs can be infused intravascularly into a patient.

There are several clinical trials utilizing RBC-based drug delivery systems (Table 1), pursuing generally two approaches. First, there is the encapsulation into RBCs of enzymes that break down specific substrates in blood. These substrates can be pathologically elevated toxic molecules (e.g., in neurological diseases) or nutrients obligatory for tumor growth. RBC-encapsulated enzymes circulate for a longer time than free enzymes and work upon substrates that diffuse from blood plasma into the loaded RBCs. Alternatively, a drug or a pro-drug encapsulated into RBCs might either circulate for a prolonged time (i.e., RBCs serve as a drug depot), or be taken up along with RBCs by phagocytes and other host defense cells, for example, for anti-inflammatory effect. The latter approach can be used for the delivery of antigens to immune system.

Encapsulation into the inner volume of RBC (A) limits interaction of the drug retained in the RBC ghost with compounds that do not diffuse through the plasma membrane; (B) helps to protect drug cargo from the body and vice versa; and (C) involves the minor fraction of RBC in blood, since this procedure takes place ex vivo, followed by the infusion of loaded RBC ghosts.

### 2.2. Surface Loading of RBCs

In the original prototypes, surface loading was achieved by chemical conjugation of cargoes to isolated RBCs ex vivo [13,14]. Newer advanced approaches grow ex vivo genetically engineered RBCs that expose specific artificial sequences, allowing subsequent site-specific biological conjugation of proteins [2]. Furthermore, nanocarriers can be adsorbed on the RBC surface non-specifically, providing formulations for RBC hitchhiking (RBC hitchhiking, see below). These iterations of RBC carrying surface-bound agents need to be infused intravascularly, similarly to RBCs loaded with drugs into the inner volume.

In several advanced iterations, drugs and carriers can be conjugated to or fused with antibodies, antibody fragments, peptides, or other ligands that bind to the RBC surface [15,16,17,18]. In this approach, RBC-targeted drugs can be used in two main protocols. First, this can be employed for ex vivo surface loading on donor or autologous RBCs, which is a multi-step, potentially damaging loading approach. Second, a simple intravascular injection of these RBC-targeted agents leads to rapid binding to RBCs circulating in the bloodstream, removing the need for any ex vivo manipulation of RBC [17].

**Figure 1:**
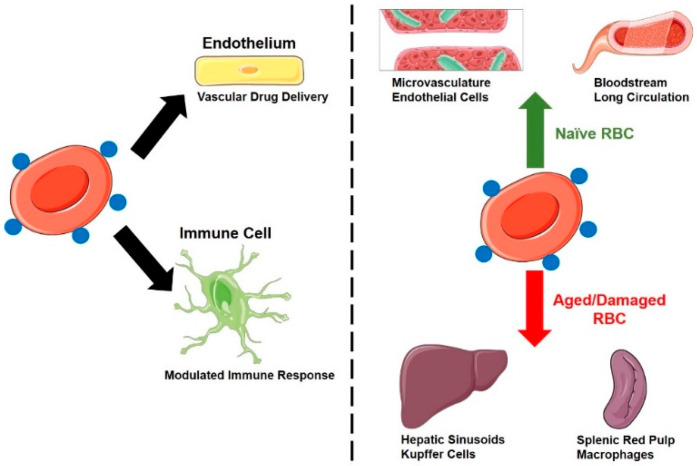
Distribution of drugs attached to the RBC surface. Left panel: Major cell types that take up surface-coupled drugs include vascular endothelium and a diverse array of immune cells (monocytes/macrophages, dendritic cells, neutrophils etc.). Right panel: RBC status (naïve vs. damaged/aged) impacts the tissue distribution of surface-coupled drugs.

Drug delivery systems based on surface loading can find utility for the prolongation and redistribution of agents that are supposed to work in the bloodstream and other sites accessible for RBCs (Figure 1), including vascular endothelium, hepatic sinuses, pathological sites of RBC diapedesis and hemorrhages, and sites of surveillance and phagocytosis of senescent RBC. These drug cargoes include drugs regulating blood fluidity, inflammation, decoy and capture systems for pathological agents, immunological reactions, and some of the applications described above for RBC-encapsulated agents [19,20,21,22].

In addition, RBC drug delivery systems can be surface-modified to confer specific affinity to therapeutic sites of interest, including those of vascular injury [13,14,23,24]. Antibody-coated RBCs have been studied in vitro and in vivo for targeting drugs to diverse cells—endothelium, smooth muscle cells, leukocytes, etc. [25,26,27]. More recently, RBCs painted with affinity ligands were tested for targeting to circulating leukocytes [28,29].

Surface RBC loading is pursued for many drugs including anti-thrombotic and anti-inflammatory agents (Table 2). For example, RBC-bound fibrinolytic agents surpass the efficacy and safety of free agents in animal models of stroke [30], traumatic brain injury (TBI) [31], hypoxia [32], and thrombosis [33] in the cerebral vasculature. Coupling to RBC carriers creates high local concentrations of tissue plasminogen activator (tPA), allowing the expedient focal dissolution of blood clots in close vicinity of the RBC-tPA complexes, which in models of thrombotic occlusion of blood vessels results in formation of patent channels spanning the plug and permitting reperfusion prior to dissolution of the thrombus [34,35].

### 2.3. RBC Loading: Effects on Cargoes

Both encapsulation into and surface-loading onto RBCs may modulate the response of immune and innate host defense system to biotherapeutic agents (see below). Both methods dramatically alter the PK of the drug cargoes.

Drug loading into RBCs physically separates cargoes from the body. Surface coupling to a large RBC carrier markedly limits the ability of drugs to interact with cognate receptors and other counterparts [43]. This mitigates the adverse effects of such interactions [44]. Furthermore, RBC-bound drugs are influenced by the RBC glycocalyx [45]. For example, RBC-coupled tPA is protected against plasma inhibitors by the RBC glycocalyx [46].

Therefore, drug loading to carrier RBCs profoundly alters their pharmacological profile, in some cases enabling effects simply unavailable to free drugs. It is tempting to postulate that RBCs may also significantly and favorably modulate the delivery and therapeutic effects of nanocarriers, among other artificial drug delivery systems, and these interactions with nanocarriers are detailed below.

RBCs have also been membrane-engineered to reduce RBC antigen immunogenicity. The ability to camouflage allogenic erythrocytes and create a universal RBC, in theory, would minimize the risk of potentially life-threatening hemolytic transfusion reactions as well as shortages of matched blood groups. Various polymer types, such as polyethylene glycol (PEG) and hyperbranched polyglycerol, have been covalently conjugated to the RBC surface [47,48]. For example, the engineering of PEG to the RBC membrane concealed glycophorin A epitopes from antibody detection and even inhibited the invasion of the malaria parasite [49]. Chronic transfusions of murine RBCs conjugated to mPEG revealed a normal in vivo circulation time with no evidence of antibody response against PEG [50]. However, excessive modification can have a deleterious impact on cell integrity and biocompatibility, and thus maintaining a balance between epitope shielding and membrane deformability is crucial [51]. More recently, investigators have developed a flexible, crosslinking nanogel barrier of polysialic acid (PSA) and tyramine to conceal RBC RhD epitopes without compromising RBC membrane structure [52].

Given the utility of polymers in RBC membrane engineering, polymers have also been employed for therapeutic surface loading. In a recent work, PEG was used to engineer immunoglobin-based therapeutics to the RBC surface [19]. The system consisted of an RBC covalently coupled to Protein A (SpA) via a PEG crosslinker. Therapeutic antibodies, specifically anti-tumor necrosis factor (anti-TNFα), were then presented onto the surface of RBCs by binding to SpA at the Fc region thus orienting the antigen-binding sites outward. The approach was rapid, cell-tolerated, and retained the functionality of the attached antibody.

### 2.4. Novel Strategies

Apart from the different advantages of RBCs mentioned above, RBCs offer unique opportunities from an engineering standpoint. First, mature RBCs are enucleated, allowing the attachment of immunomodulatory drugs, which are known to play a role in switching phenotypes, without altering physiological functions of the RBC or worsening disease pathology. This feature provides an edge over other malleable cell-based therapies such as macrophages, which can lead to disease progression due to switched phenotypes. Second, RBCs (and RBC-associated drugs) have access to every tissue in the body. Tissue-specific targeting can be achieved by optimizing a known set of parameters and the site of injection. Moreover, the versatile biomechanical properties of an RBC allow for squeezing through blood vessels of a diameter smaller than itself, creating the opportunity to target vascular endothelia of high-density capillary beds. Lastly, the ability of RBCs to negotiate immune cell clearance in the liver and spleen, until they are senescent, offers a different pathway for presenting antigens to the immune cells in these organs [53,54]. This, coupled with innate immune features of the RBC, allowing for physical adsorption of immune complexes and certain bacteria on the surface and their subsequent transfer to antigen presenting cells (APC) in the spleen, offers opportunities for immunological intervention [55,56,57]. As a result, several novel strategies are emerging to engineer RBC-DDS.

One such approach is to physically adsorb nanocarriers onto the surface of RBCs (RBC hitchhiking) [5,6,58]. Binding of nanocarriers relies on non-covalent interactions between nanocarriers and RBC membranes, including electrostatic interactions, hydrophobic interactions, and hydrogen bonding, among others. These interactions enable the successful binding of a broad spectrum of nanocarriers, ranging from synthetic nanocarriers (liposomes, polymeric nanoparticles, etc.) to natural particles (e.g., virus particles), to RBCs. In addition, nanocarrier binding to RBCs occurs across multiple species, ranging from mouse to human [6]. The binding strength and dislodging capacity of nanocarriers on RBCs can be tuned by modulating their material and surface properties.

RBC hitchhiking has been shown to significantly alter the PK profile of attached nanocarriers [6,58]. In particular, RBC hitchhiking could dramatically extend the circulation time of nanocarriers possibly due to reduced reticuloendothelial system (RES) clearance [59]. More interestingly, RBC hitchhiking significantly alters the biodistribution (BD) of nanocarriers. When administered intravenously, nanocarriers hitchhiked on RBCs exhibit significantly reduced accumulation in the liver and spleen, and drastically increased accumulation in the lung, compared to their free counterpart. The accumulation of nanocarriers in the lungs seems to be attributed to the transfer of nanocarriers to the vascular endothelium when RBCs squeeze through narrow capillaries in the lung [58]. Applying this mechanism, RBC hitchhiking was demonstrated to be able to target chemotherapeutic drug nanocarriers to lung metastasis and lead to improved anti-tumor efficacy [5]. Moreover, RBC hitchhiking could deliver nanocarriers to a wide range of organs depending on the injection sites. Specifically, hitchhiked nanocarriers exhibit enhanced accumulation in the first vascular bed encountered following their intravascular administration [6].

Another emerging approach towards creating RBC DDS is to manufacture RBCs carrying therapeutics via genetic engineering of hematopoietic precursor cells (HPCs) [60]. In this approach, CD34^+^ HPCs collected from a healthy O negative donor are genetically engineered to express one or more biotherapeutic proteins inside cells or on cell surface. These engineered cells are expanded, differentiated, and enucleated in a bioreactor to obtain the final RBCs carrying therapeutics. RBCs generated by this approach seem to have many unique properties. For example, RBCs with enzymes expressed inside cells show promise in treating metabolic diseases such as phenylketonuria (ClinicalTrials.gov Identifier: NCT04110496). In addition, RBCs with proteins expressed on cell surfaces can have specific interactions with diverse arms of the immune system, depending on the properties of the surface-expressed proteins. For example, RBCs carrying a specific set of surface proteins can activate T cells or NK cells, which are being investigated for therapy of solid tumors and leukemia [61,62,63]. The genetic engineering of RBC membranes has also been coupled with enzymatic modification. RBCs displaying sortase-modifiable membrane proteins enables the covalent, site-specific attachment of a broad range of functional probes and therapeutics [2,3]. This approach was recently used to display disease-causing antigens on RBC membranes that may be able to induce immune tolerance and is being studied for treating autoimmune diseases [3].

Previously, genetic engineering of RBCs has been employed to coat RBC membranes with viral snares. RBCs lack the key machinery necessary for viral gene expression, making them impervious to infection. In a study on coxsackievirus B, investigators engineered murine RBCs to express the virus’s target receptor protein, enabling entrapment of the virus inside the cell [64]. Mice expressing the modified RBCs demonstrated curtailed viremia and lowered viral proliferation in vital organs. With the emergence of Coronavirus disease 2019 (COVID-19), extensive efforts have been made in developing novel therapies to combat this pandemic, such as the conjugation of viral snares to RBCs. However, the most apparent obstacle to this approach is the necessity to genetically engineer erythroid precursors. The lengthy process of RBC in vitro generation limits the production rate and availability of this therapeutic delivery system. A more rapid and tunable chemical engineering strategy would be a more viable approach to couple the virus’s binding-ligands to the RBC surface.

## 3. Pharmacokinetics of RBC-Associated Drugs

### 3.1. Pharmacokinetics—A Brief Primer

#### 3.1.1. Absorption and Routes of Administration

Following extravascular administration of therapeutics, there are a number of barriers that drugs must overcome in order to enter the systemic circulation [65,66]. These processes governing the entry of drug into the circulation are typically described under the umbrella of “absorption”. The primary metrics that are used to describe absorption relate to the rate and extent of absorption are the maximum observed blood concentration (*C*_max_), time of *C*_max_ (*t*_max_), and bioavailability (*F*) (Table 3). As extravascular administration is not an option for drugs carried by erythrocytes, we will only provide a brief summary of pharmacokinetic expectations following extravascular administration to facilitate comparisons. Select routes of administration and associated pharmacokinetic expectations are summarized in Table 4.

#### 3.1.2. Distribution

**Figure 2:**
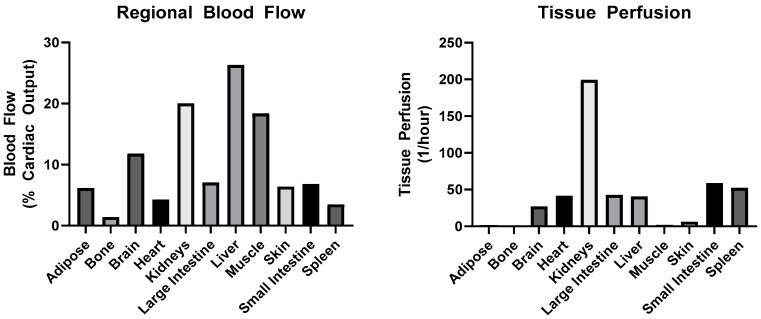
Regional blood flow and relative tissue perfusion in healthy humans. Values obtained from [70].

Following the entry of the drug into the systemic circulation, the drug is able to move between blood and tissues, in a process termed distribution. The primary metric that is used to describe the extent of drug distribution is the volume of distribution at steady state (*V*_ss_) (Table 3). It should be noted that *V*_ss_ does not represent any physical volume, but it does have a lower limit of plasma volume. Barriers to tissue distribution include: (1) tissue perfusion, (2) diffusion, (3) protein/cell binding, (4) active transport, and (5) vascular permeability. Tissue perfusion is largely governed by blood flow to the tissue. In Figure 2, plots of regional tissue blood flow and perfusion are shown for a typical adult human. All else being equal, the greater the fractional cardiac output delivered to the tissue, the greater the chance for distribution into the tissue. Of note, the lung is not depicted, as it has a unique circulation, receiving 100% of venous output from the heart via the pulmonary arteries and a fraction of arterial output via the bronchial circulation. The only other tissue that receives venous blood is the liver, which collects blood both through the hepatic artery and the portal vein, which drains from the intestines, spleen, and pancreas. A summary of primary mechanisms of tissue distribution is provided in Table 5 to facilitate comparisons between different classes of drug molecules.

#### 3.1.3. Metabolism/Elimination

The removal of an active drug substance from the circulation can occur either as direct elimination of the unchanged drug or via metabolism of the drug molecule and subsequent elimination of the metabolite. The primary metric used to describe the efficiency of elimination is clearance (CL), which describes the volume from which drug is removed per unit time. While there is no theoretical lower bound on clearance, the upper limit to this parameter is the sum of blood flows to the eliminating organs. A summary of key routes of elimination for a variety of drug classes is provided in Table 6.

#### 3.1.4. Pharmacokinetics in Drug Development

Proper characterization and understanding of PK properties of drug candidates is critical for effective drug development. In the early 1990s, poor understanding of pharmacokinetics early in development resulted in PK being the most common reason for attrition (40%) during clinical trials; however, by the end of the decade, this had fallen to less than 10% of failures, as early, thorough characterization of PK became more common [76]. Additionally, as drugs move through clinical development and onto the market, population pharmacokinetics is used to describe the impact of patient-related factors (termed covariates) on in vivo behavior [77]. For example, between 2003 and 2017, 94% of monoclonal antibodies that were approved by the FDA included population PK analysis, often relating factors such as body size, gender, and renal function to mAb PK [78]. By characterizing PK, not only at the level of the population, but also at the level of the patient, individualized dosing becomes feasible. Ultimately, thorough understanding of pharmacokinetics, and the factors influencing it, permits the development of drugs with a higher probability of favorable safety and efficacy profiles.

#### 3.1.5. ADME of RBC-Associated Drugs

Regardless of the method of attachment of drugs to carrier erythrocytes, proper analysis of PK is complicated by the number of potential analytes. Complete characterization of PK would include measurements of RBC-associated drug, released drug (plasma or serum), and survival of the infused RBCs. As summarized in Table 7, despite significant interest over several decades in the use of RBCs for prolonged drug delivery, there is relatively little standardization in bioanalytical measurements. Nonetheless, RBC encapsulation generally leads to improvements in PK relative to free drugs and enhanced efficacy both in animal models and in patient populations. 

#### 3.1.6. Unique Aspects of RBC PK

As highlighted above (Table 5 and Table 6), PK expectations for RBCs (and drugs carried by them) are distinct from those for small molecules, biologics, and even DDS. This is in no small part due to their large size (6–8 μm vs. 100–200 nm for DDS), flexibility, and coating with specific markers that prevent their premature clearance by the immune system (e.g., CD47) [105]. Under normal conditions, RBCs will be entirely confined to the bloodstream, making the only feasible route of administration for RBC-carried drugs via intravascular routes (e.g., intravenous and intra-arterial). This is a stark contrast from other classes of drug, which are able to be given via many routes of administration (Table 3). This exclusive localization in the bloodstream will minimize tissue uptake of RBC-associated drugs, potentially eliminating off-target toxicities.

**Figure 3:**
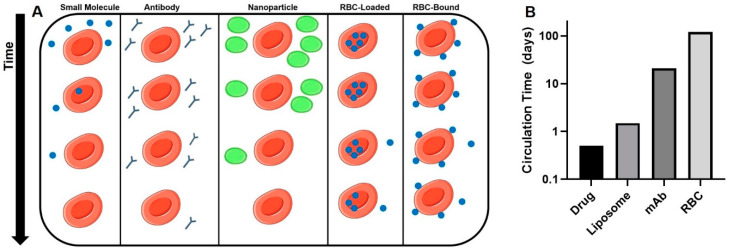
Pharmacokinetics of various drug delivery strategies. (**A**): Circulation time and intravascular distribution of drugs administered in free form, as antibodies, in nanoparticles, and associated with RBCs. (**B**): Typical circulation time in humans of drugs and drug delivery systems (DDS). Approximate circulation times for liposomes [106], mAbs [107], and RBC [21].

Immediately after injection of RBC-associated drugs, the drug will be exclusively localized within the cellular component of blood (Figure 3A). This is in stark contrast to other classes of drugs, which, in the absence of specific affinity moieties, are largely localized in the plasma. However, the system is dynamic, and as time progresses, the drug will slowly leak out of the RBC until a pseudo-equilibrium between cells, plasma, and tissues is reached. The rate and extent of RBC leakage is likely to be unique to each drug and method of coupling/encapsulation.

Most small molecule and protein therapeutics are eliminated from the circulation with half-lives of minutes to hours, necessitating frequent dosing. While this may not be a significant concern for drugs with good bioavailability and/or wide therapeutic indices, this is often a limiting factor in efficacy. Approaches to extend the circulation time and to improve targeted delivery of therapeutics, such as encapsulation in liposomes (hours–days) and conjugation to mAbs (days–weeks), have been applied clinically with some success [108,109]. However, there is no method for improving PK approaches the potential of coupling to RBCs, which circulate with a lifespan of 100–120 days in adult humans (Figure 3B). This unprecedented circulation time offers great potential for the use of RBCs as a slow release depot to obtain constant concentrations of active drug in the body.

As summarized in Table 6, small molecule drugs are typically eliminated either via the kidney (glomerular filtration or active tubular secretion) or by active metabolism (e.g., Cytochrome P450s). While a drug that leaves the carrier RBC will be subjected to these traditional clearance pathways, an encapsulated drug is subject to the disposition properties of the RBC. The elimination of RBCs is under the control of phagocytic cells in the reticuloendothelial system (RES). These cells, particularly in the splenic red pulp under normal conditions, sense damaged and aged RBCS and eliminate them from the circulation. Co-opting of this clearance pathway by RBC-associated drugs would be expected to favor uptake of drug by phagocytes.

### 3.2. PK of RBC-Associated Drugs—Key Considerations

Thorough analysis of PK of RBC-associated drugs requires information describing the blood and tissue PK of the RBC-associated drug, released drug, and carrier RBCs. While this degree of characterization may seem daunting, in reality it only requires two distinct bioassays, typically liquid chromatography-mass spectroscopy for small molecule drugs and a method to trace infused RBCs (e.g., ^51^Cr labeling).

#### 3.2.1. Drugs Loaded inside the RBC—Effects on RBC Circulation

A common approach for loading RBCs with both small molecule drugs and protein therapeutics is to swell the RBC in hypotonic buffer containing the drug, favoring entry of the drug into the RBC, followed by returning the RBC to an isotonic buffer, trapping the drug inside the RBC. This process has been shown to have adverse effects on RBC circulation time compared to ‘naïve’ RBCs in mice [97] and in rats [84]. Nonetheless, the half-life of resealed erythrocytes (~10 days in mice following L-asparaginase loading [97]) is still much greater than what would be anticipated for other DDSs (e.g., liposomes). In the same study, it was shown that increasing the concentration of L-asparaginase used for RBC loading did not have a large impact on RBC circulation time, but rather the swelling and resealing process caused the majority of changes in RBC circulation. This is suggestive that while circulation of carrier RBCs that had been loaded through a potentially damaging process (e.g., hypotonic preswelling) is likely to be reduced, these carriers still present a viable option to improve the circulation time of drugs far beyond that which is typically obtained with DDS.

#### 3.2.2. Drugs Loaded Inside the RBC—Effects on Drug Pharmacokinetics

**Figure 4:**
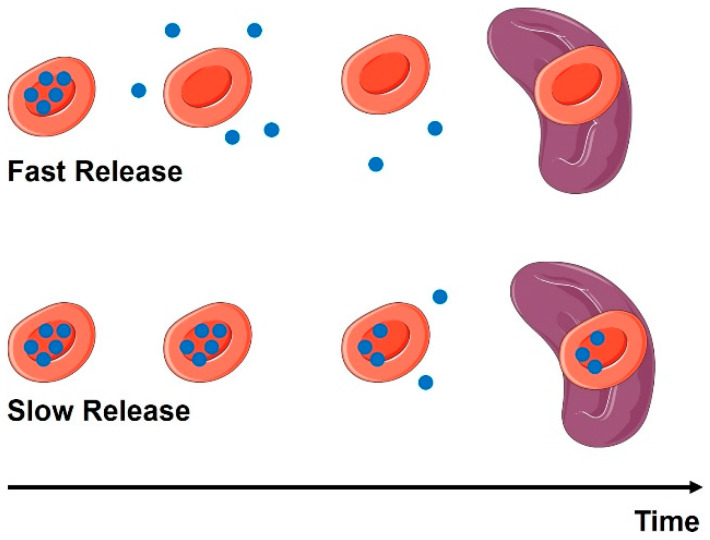
Impact of release kinetics on drug disposition. Upper panel: Rapid release from the RBC will lead to dose dumping in plasma and separate elimination of the drug and RBC. Lower panel: Slow release of the drug will lead to some of the drug being released in plasma and some being taken up into clearance organs (e.g., spleen, depicted) with the RBC. Blue symbols represent drug molecules loaded into a carrier erythrocyte. Over time, the drug leaks from the cell with distinct kinetics (either fast or slow), affecting the biodistribution of the drug.

Once loaded into the RBC and infused into patients, the drug will initially be entirely localized within the RBC fraction of blood. In many cases, the goal of RBC loading is to provide a long circulating depot of drug that will be slowly released from the RBC. As such, the primary driver of free drug exposure would be the rate of release from the RBC. Release kinetics have been broadly defined based on the release relative to hemoglobin, which is used as a marker of hemolysis [110]. For drugs that are released quickly from the RBC, diffusion is the likely explanation. These molecules are typically relatively small and hydrophobic, permitting diffusion across the cell membrane. Drugs that are released very slowly (similar kinetics to hemoglobin) are thought to only escape the RBC following the destruction of the cell, and as such are typically polar drugs and proteins. Knowledge of release kinetics permits identification of likely sites of release and drug PK following release (Figure 4). Release from intact RBCs would likely manifest as a slow appearance of drug in the plasma, similar to what might be expected from an IV infusion. Following release, the free drug would then follow its usual distribution, metabolism, and elimination processes. On the other hand, for drugs that are not released until the RBC is lysed, it is expected that there will be minimal appearance in plasma, but rather, drug will appear within RES organs (spleen and liver). However, to have any effects beyond local, intracellular response, the drug would need to be able to escape from phagocytic cells and natural clearance pathways for drugs (e.g., CYP450s). In this case, one would expect that drug PK would largely follow RBC PK, with differences potentially occurring post-RBC lysis.

#### 3.2.3. Surface Loading of RBC—Impact of Dose and Coupling Strategy

Another approach utilized for RBC-mediated drug carriage is to couple drugs to the cell surface either via covalent binding, formation of a streptavidin-biotin bridge (or similar), or antibody binding to RBC membrane proteins [2,17,18,36,111,112]. A general expectation for this method of coupling would be that as the mass of drug added/RBC is increased, the circulation time would decrease due to adverse effects on the RBC membrane and/or enhanced immune recognition of the RBC. For example, it was shown through tracing of ^51^Cr-labeled RBCs that increasing the degree of RBC biotinylation led to dramatic changes in the PK of biotinylated and streptavidin conjugated RBCs [111]. While it may be tempting to speculate that this effect may be simply due to ex vivo manipulation of RBCs and covalent modification of membrane proteins, others have shown that the injection of the anti-glycophorin A mAb, Ter119 results in dose-dependent reductions in the total RBC pool [113].

However, it should be noted that the relative potency of a given coupling strategy on RBC circulation will likely be highly dependent on the method of coupling. For example, at equivalent loading doses of Ter119 mAb and its (Fab)_2_ fragment, the mAb caused more rapid elimination of coated RBCs, likely due to the presence of the Fc fragment [16]. This serves to highlight that even when binding the same epitope on the RBC surface, other molecular factors may impact RBC circulation. Differences in effects on RBCs by coupling approaches can be further exacerbated when comparing between different epitopes. For example, our group recently showed that scFv-thrombomodulin fusion proteins directed against human RhCE did not impact RBC rigidity and fragility, while those targeted to glycophorin A significantly increased these markers of RBC damage [114]. This suggests that features of the molecule used to couple to the surface of the RBC (e.g., avidity, Fc fragment, epitope) have a significant impact on the biocompatibility of the loading strategy.

#### 3.2.4. Surface Loading of RBC—Impact of Affinity

Increasing the affinity for target epitopes is generally expected to increase residence time at the target in the absence of confounding factors (e.g., internalization, target cell clearance, etc.), largely due to reductions in the rate of dissociation from the target. Due to their lack of endocytic capacity and extremely long circulation time relative to most (if not all) drugs, RBCs present an attractive system to study this phenomenon. It is clear that providing a molecule that previously had negligible affinity for RBCs with nanomolar or better affinity for an RBC membrane protein is capable of generating remarkable improvements in PK. Our group has previously demonstrated that coupling plasminogen activators (PA, *t*_1/2_ ~ minutes) to RBCs via affinity binding [18] or streptavidin-biotin coupling [36] permits PA to circulate with a half-life on the order of days in mice. While this example provides clarity on what happens at an extreme end of the affinity spectrum, there are no reports on what happens at intermediate affinity values.

Beyond simply considering equilibrium affinity for the surface of RBC, the kinetics of association and dissociation can be critical in determining the circulation time and biodistribution of surface-coupled therapeutics. By tuning these parameters, one can gain tighter control over the behavior of therapeutics. For example, to maximize circulation time, it would be expected that an affinity ligand with a rapid association rate and slow dissociation rate would be ideal, as it would favor prolonged binding to the RBC. On the other hand, if a therapeutic rapidly dissociates from the RBC surface, it would have the opportunity to rapidly distribute into tissues immediately downstream of the injection site. This phenomenon underlies a recent development from our group—RBC hitchhiking. In this approach, nanoparticles adsorbed (with no affinity ligand) onto the surface of RBCs ex vivo are taken up rapidly and in large numbers in the first vascular bed encountered following injection (e.g., the lung following IV dosing) [5,6,7,115]. Gaining an understanding of what range of equilibrium affinities and on-/off-kinetics provide PK benefits without adversely affecting the RBC is critical in further characterizing this method of drug loading and developing clinically viable strategies for surface loading of RBC.

#### 3.2.5. Surface Loading—Ex Vivo or In Vivo?

A key advantage to using affinity ligands to attach a therapeutic to the surface of RBCs is the potential for in vivo loading, as compared to covalent conjugation and internal loading, which require ex vivo manipulation of RBCs and reinfusion. The loading of drugs in vivo could greatly reduce the possibility of damaging RBCs, leading to rapid elimination or severe toxicities related to hemolysis. It is important to consider how the location of loading could affect PK of carrier RBCs and associated drugs.

**Figure 5:**
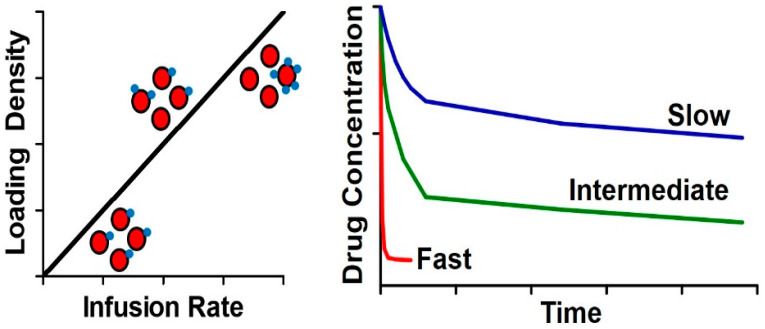
Infusion rate impacts in vivo behavior. Left panel: Relationship between infusion rate and loading density on RBCs. Right panel: Impact of infusion rate/loading density on circulation time of RBC-associated drugs.

Ex vivo manipulation and loading of RBCs, followed by reinfusion into patients, will result in the entire injected dose being present on a small fraction of total RBCs in the bloodstream. On the other hand, in vivo loading allows tuning the degree of RBC loading by adjusting the infusion rate. For example, if the dose is administered very quickly, as an IV bolus, loading will be similar as what would be expected for ex vivo loading. However, a very slow IV infusion, over minutes to hours, would provide a relatively lower load on each individual RBC, but a more homogeneous distribution across the entire RBC population, potentially leading to fewer adverse effects on the RBCs, and longer circulation times (Figure 5).

## 4. Conclusions and Other Perspectives

For almost 50 years, academic researchers have been intrigued by the potential of using autologous RBC as a drug delivery system for a myriad of therapeutics. The initial concept of loading enzymes into the RBC has been extended and is now applied to a diverse range of loading strategies (intracellular, surface coupled) and therapeutic modalities (small molecules, protein therapeutics, nanoparticles). Attachment of drugs to the RBC can lead to unprecedented changes in circulation time, biodistribution, elimination pathways, pharmacodynamics, and immunogenicity. Therapeutics that are attached to the surface of the RBC are able to directly interact with and transfer to the endothelium, particularly within capillaries where the RBC is deformed and squeezes through the vessel. Recent advances have allowed this field to move from an academic curiosity into a potentially clinically used strategy. These include approaches for rapid ex vivo loading of RBC, development of new affinity ligands for coupling to membrane proteins, and genetic engineering of the RBC. Despite these significant technological advances, there is a clear gap of knowledge in many areas related to the PK/BD of RBC-associated drugs that we hope to begin to close through careful investigation of the unique aspects of RBC PK that we have described.

## Figures and Tables

**Table 1:** Summary of clinical trials for red blood cell (RBC)-associated drugs.

Company	Drug	Disease	Trial Identifier
EryDel	Dexamethasone	Ataxia Telangiectasia	NCT03563053
Erytech	L-Asparaginase	Triple-Negative Breast Cancer	NCT03674242
		Acute Lymphoblastic Leukemia	NCT03267030
		Pancreatic Ductal Adenocarcinoma	NCT03665441
Anokion	KAN-101	Celiac Disease	NCT04248855
Rubius	Phenylalanine Ammonia Lyase	Phenylkentonuria	NCT04110496

**Table 2:** Approaches for coupling drugs to the RBC surface.

Coupling Strategy	Drug	Indication	References
Streptavidin-Biotin	tPA	Pulmonary Embolism	[36,37]
		Arterial Thrombosis	[36]
		Thrombotic Stroke	[30,38]
		Traumatic Brain Injury	[31]
		Cerebral Hypoxia	[32]
Antibody (or Fragment) Binding	tPA	Pulmonary Embolism	[39]
		Arterial Thrombosis	[39]
		Pulmonary Embolism	[18]
	Reteplase	Venous Thrombosis	[40]
	scuPA-T	Cerebral Thrombosis	[33]
	Thrombomodulin	Vascular Thrombosis	[17]
		Endotoxemia	[20]
		Cerebral Ischemia/Reperfusion	[20]
Peptide Binding	Protein Antigens	Immune Tolerance Induction	[41,42]
Passive Adsorption	NP-Reteplase	Pulmonary Embolism	[6]
	NP-Doxorubicin	Lung Metastasis	[5]

**Table 3:** Key pharmacokinetic parameters.

Parameter	Definition
Area Under the Curve (AUC)	Primary metric of overall drug exposure
Terminal Half-Life (*t*_1/2_)	Time for drug concentrations to reduce by 50% during the terminal slope (*λ*_z_)
Maximum Blood Concentration (*C*_max_)	Highest observed blood concentration
Time of *C*_max_ (*t*_max_)	Time post-dosing where *C*_max_ occurs
Bioavailability (*F*)	Fraction of administered dose that reaches the systemic circulation
Clearance (CL)	Volume cleared of drug per unit time
Mean Residence Time (MRT)	Average time that a drug molecule stays in the body
Volume of Distribution (*V*_ss_)	Relationship between the amount of drug in the body and the blood concentration

**Table 4:** Pharmacokinetic Expectations for Select Routes of Administration.

Route	Typical *t*_max_	Barriers	Advantages	Clinical Use
Oral	Variable ^a^	Harsh GI environment Efflux transporters First-pass metabolism	Safe and painless Patient convenience	Small molecule
Subcutaneous	Hours–days [67]	Immune system	Patient convenience	Peptides
			No first-pass	Proteins
Inhaled	Seconds–minutes [68]	Airway branching	Local delivery	Small molecule
		Muco-ciliary clearance	Rapid absorption	
		Immune system	No first-pass	
Transdermal	Hours–days [69]	Dense layers of skin and fat	Prolonged delivery	Small molecule
		Immune system	No first-pass	

^a^ The rate of drug absorption after oral administration is highly variable and dependent on drug, subject, and dosage form-related factors.

**Table 5:** Mechanisms of tissue distribution.

Drug Class	Mechanisms	Barriers
Small Molecule	Diffusion	Plasma protein binding
	Uptake transporters [71]	Efflux transporters [71]
Peptides/Proteins	Diffusion (Low MW)	Vascular permeability [72]
	Bulk fluid flow Receptor-mediated transcytosis [73]	
Drug Delivery Systems	Bulk fluid flow Receptor-mediated transcytosis [73]	Vascular permeability [72]
Erythrocytes	N/A	Vascular permeability [72]

**Table 6:** Primary routes of elimination.

Drug Class	Mechanisms	Primary Tissues
Small Molecule	Renal filtration	Kidney
	Active tubular secretion	Kidney
	Metabolism [74]	Liver, GI, etc.
Peptides/Proteins	Renal filtration (<60 kDa)	Kidney
	Non-specific catabolism	Liver, spleen, etc.
	Receptor-mediated clearance	Target tissue
Drug Delivery Systems [75]	Immune cell uptake	Liver, spleen
	Receptor-mediated clearance	Target tissue
Erythrocytes	Macrophage uptake	Spleen, liver

**Table 7:** Summary of reported RBC-associated drug pharmacokinetic parameters.

Drug	Species	Condition	PK Changes	Pharmacologic Effect (Relative to Free Drug)	References
5-Fluoruracil (5-FU)	Mouse	Malignant Ascites	2-fold increase in AUC_0-inf_ in ascites fluid	70% survival at 20 days vs. 20% in malignant ascites model	[79]
Adenosine Deaminase (ADA)	Human	ADA Deficiency	2–4-fold increase in ADA t_1/2_ 57-day lifespan of loaded RBC		[80]
Alcohol Dehydrogenase (ADH) Aldehyde Dehydrogenase (ALDH)	Mouse	Healthy	4.5-day RBC t_1/2_	43% reduction in blood ethanol concentrations vs. empty RBC	[81]
Amikacin	Rat	Healthy	2-fold increase in AUC_0-inf_ in plasma Large increases in liver/spleen AUC_0-inf_		[82,83]
Carbonic Anhydrase	Rat	Healthy	Similar circulation time as carrier RBC 9-day t_1/2_ of loaded RBC		[84]
Daunorubicin	Human	Acute Leukemia	~2-fold increase in blood t_1/2_		[85]
Dexamethasone	Human Human Human Rabbit	Inflammatory Bowel Disease Chronic Obstructive Pulmonary Disease Cystic Fibrosis Healthy	Plasma concentrations detectable 28 days post-infusion Plasma concentrations detectable for at least 1 week post-infusion Relatively constant plasma concentrations for at least 10 days ~60-fold increase in plasma *t*_1/2_	50% reduction in ESR and CRP relative to standard of care Reduction in ‘as-needed’ use of corticosteroids and β-agonists Improved FEV_1_ and 51% reduction in antibiotic use Reduction in histamine response	[86,87,88,89,90]
Doxorubicin	Human	Lymphoma	2-7-fold increase in plasma *t*_1/2_		[91]
Erythropoietin	Mouse	Healthy	~5-fold increase in blood AUC 5.6 day RBC *t*_1/2_	~2-fold increase in ^59^Fe incorporation into circulating RBC	[92]
Factor IX	Human	Healthy	~8-fold increase in blood t_12_		[93]
Gentamicin	Human	Healthy	22 day blood t_1/2_		[94]
Imidocarb	Mouse	Parasitemia	Significantly increased blood concentrations	~25% reduction in peak parasitemia	[95]
Indinavir	Rat	Healthy	9-fold increase in plasma AUC_0-inf_		[96]
L-Asparaginase	Mouse Mouse Mouse	Healthy Healthy Acute Lymphoblastic Leukemia	~3-fold increase in blood *t*_1/2_ 9–10.6 day RBC *t*_1/2_ 16-fold increase in blood *t*_1/2_ 2.4–4 day blood *t*_1/2_	4–5-fold increase in duration of maximal asparagine lowering >10-fold increase in duration of total asparagine suppression Reduced ADA formation 44% increase in survival time vs. untreated	[42,97,98]
Maltose-Binding Protein	Mouse	Healthy	~3-fold increase in blood t_1/2_		[15]
Methotrexate	Mouse	Healthy	3.5-fold increase in plasma *t*_1/2_ ~2-fold increase in liver and spleen uptake		[99]
Phenylalanine Hydroxylase	Mouse	Naive	Detectable drug in blood for at least 10 days post-injection vs. <6 h	~50% reduction in blood Phe vs. 25%	[100]
Prednisolone	Rat	Healthy	High drug uptake in liver		[101]
Polystyrene Nanoparticles	Mouse	Healthy	2–3-fold increase in blood exposure ~5-fold increase in lung uptake >50% decrease in spleen uptake No effect on RBC survival (*t*_1/2_ = 33.5 h)		[58]
Reteplase	Mouse	Acute Thrombosis	Blood *t*_1/2_ of ~10 h vs. minutes No impact on RBC circulation time	~3-fold delay in time to arterial occlusion Complete prevention of venous occlusion	[18]
Rhodanese	Mouse	Healthy	230-fold increase in *t*_1/2_	40% reduction in blood cyanide following IV injection	[102]
Tissue Plasminogen Activator	Mouse Rat	Acute Thrombosis Acute Thrombosis	~10-fold increase in blood exposure No changes in RBC survival >10-fold increase in blood AUC Minimal effects on RBC circulation	~50% lysis of pulmonary emboli Significant reduction in mortality from thromboembolic stroke ~80% lysis of pulmonary emboli ~80% of blood flow recovery in carotid artery	[30,31,32,36,38,39,103]
Thrombomodulin	Mouse	Acute Thrombosis Ischemic Stroke Endotoxemia	10% of drug present in blood 2 days post-injection vs. 1 h No changes in RBC survival	Complete protection against jugular vein thrombosis ~50% reduction in infarct volume and neurological deficit >50-fold improved potency at reduction of pro-inflammatory cytokines	[17,20]
Urokinase	Rabbit Mouse	Healthy Acute Thrombosis	Significant increase in blood exposure 14-fold increase in blood concentration at 30 min No changes in RBC survival	4–5-fold increase in blood flow following carotid artery thrombosis ~3-fold increase in blood flow following venous thrombosis	[33,40,104]

Notes: Unless otherwise noted, comparisons of PK/PD measurements are relative to free drug. Unless explicitly stated as being RBC related (e.g., RBC survival), all measurements relate to the PK of the therapeutic payload. Abbreviations used in table: AUC: area under the concentration vs. time curve, ESR: erythrocyte sedimentation rate, CRP: C-reactive protein, FEV1: forced expiratory volume, ADA: anti-drug antibody, Phe: phenylalanine.
